# Transcranial doppler assessment of cerebral perfusion in critically ill septic patients: a pilot study

**DOI:** 10.1186/2110-5820-3-28

**Published:** 2013-08-22

**Authors:** Charalampos Pierrakos, Aurélie Antoine, Dimitrios Velissaris, Isabelle Michaux, Pierre Bulpa, Patrick Evrard, Michel Ossemann, Alain Dive

**Affiliations:** 1 Department of Intensive Care, Université Catholique de Louvain, Mont-Godinne University Hospital, Avenue Docteur G., Thérasse 1, Yvoir 5530, Belgium; 2 Department of Neurology, Université Catholique de Louvain, Mont-Godinne University Hospital, Avenue Docteur G., Thérasse 1, Yvoir 5530, Belgium; 3 Department of Internal Medicine, University Hospital of Patras, Patras Rio 26504, Greece

**Keywords:** Encephalopathy, Sepsis, Cerebral vasoconstriction, Cerebral microcirculation, Pulsatility index, Resistance index

## Abstract

**Background:**

The aim of this study is to evaluate the feasibility and efficacy of Transcranial Doppler (TCD) in assessing cerebral perfusion changes in septic patients.

**Methods:**

Using TCD, we measured the mean velocity in the middle cerebral artery (VmMCA, cm/sec) and calculated the pulsatility index (PI), resistance index (RI) and cerebral blood flow index (CBFi = 10*MAP/1.47^PI^) on the first day of patients’ admission or on the first day of sepsis development; measurements were repeated on the second day. Sepsis was defined according to standard criteria.

**Results:**

Forty-one patients without any known neurologic deficit treated in our 24-bed Critical Care Unit were assessed (Sepsis Group = 20, Control Group = 21). Examination was feasible in 91% of septic and 85% of non-septic patients (p = 0.89). No difference was found between the two groups in mean age, mean arterial pressure (MAP) or APACHE II score. The pCO_2_ values were higher in septic patients (46 ± 12 vs. 39 ± 4 mmHg p < 0.01). No statistically significant higher values of VmMCA were found in septic patients (110 ± 34 cm/sec vs. 99 ± 28 cm/sec p = 0.17). Higher values of PI and RI were found in septic patients (1.15 ± 0.25 vs. 0.98 ± 0.16 p < 0.01, 0.64 ± 0.08 vs. 0.59 ± 0.06 p < 0.01, respectively). No statistically significant lower values of CBFi were found in septic patients (497 ± 116 vs. 548 ± 110 p = 0.06).

**Conclusions:**

Our results suggest cerebral vasoconstriction in septic compared to non-septic patients. TCD is an efficient and feasible exam to evaluate changes in cerebral perfusion during sepsis.

## Background

Sepsis-associated encephalopathy (SAE) may develop in more than 50% of septic patients [1,2]. It is one of the most common causes of delirium in intensive care units [3], where it is an independent prognostic factor for increased mortality [4]. Additionally, it is suspected of contributing to long-term cognitive impairment [5].

The pathogenesis of SAE remains unclear. Alterations in cerebral perfusion during sepsis possibly play an important role in the development of this clinical entity [6]. Microcirculatory dysfunction and dissociation between cerebral cells’ needs and perfusion at several cerebral areas was found in an experimental sepsis model [7,8]. However, in humans, microcirculatory dysfunction is not widely assessed, as, to date, no widely applicable method exists to evaluate cerebral perfusion [9]. Existing attempts to evaluate cerebral blood flow and microcirculation in humans during sepsis are limited to a small number of selected patients [10,11]. Transcranial Doppler (TCD) is a readily available and reproducible technique by which cerebral perfusion can be evaluated in everyday clinical practice. Indirect cerebral microcirculation assessment by testing cerebral autoregulation in response to several stimulations with TCD has been previously performed [12-17]. However, these methods are relatively complicated and not easily applicable. The aim of this study is to assess static cerebral perfusion characteristics and changes in septic versus non-septic critically ill patients.

## Methods

This is a prospective observational study that was conducted in our 24-bed intensive care unit during a three-month period (July 2011 to September 2011). The study group consisted of 20 consecutive patients who developed sepsis in a period of 48 hours after their admission (sepsis group). Sepsis was defined according to standard international criteria [18]. We also enrolled 21 patients without any signs of infection on their ICU admission who were expected to stay in the ICU for more than 24 h (Control Group). The Ethics Committee of Mont-Godinne University Hospital approved the study protocol and verbal consent was obtained from all patients or from relatives in cases where the patient was not conscious.

Exclusion criteria for both groups were as follows: 1) age < 18 years old, 2) known cerebral lesion (ischemic or haemorrhagic cerebrovascular event, neoplasm), 3) cerebral infection, 4) encephalopathy associated with hyperuremia, hypernatremia or hypoglycaemia, 5) hepatic encephalopathy, 6) patient supported by Intra-Aortic Balloon Pump or by ECMO, 7) non-sinusal rhythm, 8) known severe carotid stenosis (>70%), or 9) history of extended cervical operation.

Demographic data on all patients and diagnosis on ICU admission were recorded. The source of sepsis, relevant microbiological results, and treatments, including administration of adrenergic and sedative agents, were recorded. The neurology status was evaluated from GCS. For the septic patients who were intubated or nonseptic patients who were intubated urgently, the GCS before the administration of sedatives was registered. For the nonseptic patients who were intubated electively, the GCS was evaluated 6 h after the sedation cessation. For the rest of the patients, the GCS on the first day of inclusion in the study was recorded. The severity of critical illness was assessed from the Acute Physiology and Chronic Health Evaluation (APACHE) II score.

Mean velocity in the middle cerebral artery (VmMCA) was measured using a 2-MHz TCD probe through the temporal bone window on both sides of the skull, twice within the first 48 h after the confirmation of sepsis diagnosis for the septic group or after ICU admission for patients in the control group. An interval period of more than 20 h between the two measurements was ensured. Each measurement on each side of the brain was repeated three times and the highest value was considered for our analysis. The average of the two values on the two brain sides was registered. A difference in depth of 0.5 cm between the two sides was considered acceptable. At the time of the measurements, the patients were in a stable hemodynamic status. Pulsatility index (PI) (PI = Velocity systolic-Velocity diastolic/mean velocity) [19] and Cerebrovascular resistant index (RI) (RI = Velocity Systolic–Velocity diastolic/Velocity Systolic) [20] were calculated. Previous experimental data showed a strong inverse correlation between PI and the logarithm of regional cerebral blood flow measured by laser Doppler (r^2^ = 0.81) [21]. From the formula that was provided (logCBF = 1.11-0.17*PI), we found that the antilogarithm of CBF was related to the PI with the following formula CBF = 10/1.47^PI^. Based on this formula, and given that cerebral blood flow (CBF) is estimated by the formula Perfusion Pressure/cerebrovascular resistances, we considered PI relate to cerebral resistances (CBVR) accordingly: CBVR ≈ 1.47^PI^/10. We evaluated the ratio MAPx10/1.47^PI^ as an index of CBF.

The primary endpoint of the study was the identification of any differences in the values of VmMCA and/or PI and/or RI in septic patients compared to non-septic critically ill patients.

### Statistical analysis

Statistical analysis was performed with SPSS software (SPSS Inc., Chicago, IL). A Kolomogorov-Smirnov test was used to verify the normality of distribution of continuous variables. Student’s *t* test was used for continuous variables. Categorical variables were compared by Fisher’s exact test. Pearson’s correlation was applied to evaluate the relationships between VMCA, PI, RI, and pCO_2_ or MAP and between VMCA and CBFi. Statistical significance was defined as *p* < 0.05.

## Results

Twenty patients with sepsis and 21 patients without any proven sign of infection were included in the study; 1 patient in the control group refused the examination. The TCD evaluation was not feasible because of a lack of a satisfactory acoustic window in 2 patients in the sepsis group and 3 patients in the control group (feasibility 91% vs. 85%, *p* = 0.89). The demographic characteristics of the assessed patients are shown in Table 1. No statistically significant differences between septic and nonseptic patients in terms of age (67 ± 11 vs. 67 ± 14 years, *p* = 0.99) or APACHE II score (21 ± 7 vs. 20 ± 5, *p* = 0.92) were observed. The mean arterial pressure was not found to be different between the two groups (77 ± 14 vs. 79 ± 14 mmHg, *p* = 0.51), whereas the percentage of patients who were evaluated for treatment with norepinephrine at a dose > 5 μg/min was similar for the two groups of patients (30% vs. 22%, *p* = 0.56). The pCO_2_ values were higher in septic patients compared with controls (46 ± 12 vs. 39 ± 4 mmHg, *p* < 0.01).

**Table 1:** Demographic and hemodynamic characteristics of the patients

	**Sepsis group**	**Control group**	***p values***
No patients	18	18	
Age (yr)	67 ± 14	67 ± 11	0.99
Gender (male)	11(61%)	13 (72%)	0.72
Coronary Disease	5 (27%)	11 (61%)	0.03
Type of patients			
*Medical*^*a*^	11 (61%)	4 (22%)	0.04
Medications^b^			
*Norepinephrine*^*c*^	12 (33%)	10 (28%)	0.79
*Dobutamine*^*d*^	4 (11%)	10 (28%)	0.05
*Sedation*^*e*^	13 (33%)	16 (44%)	0.63
APACHE II	21 ± 7	20 ± 5	0.92
GCS < 14^f^	11 (61%)	3 (16%)	0.03
GCS^f^	9 ± 5	14 ± 1	0.08
MAP (mmHg)	77 ± 14	79 ± 19	0.51
Hg (g/dl)	9.4 ± 1.4	10.1 ± 1.4	0.36
pCO_2_ (mmHg)	46 ± 12	39 ± 4	<0.01
pH	7.39 ± 0.09	7.37 ± 0.05	0.58

*GCS*, glasgow coma score; *MA,* mean arterial pressure; *Hg*, hemoglobin.

^a^Medical patients: patients not operated on before their evaluation;^b^Number of measurements under these medications;^c^ >0.06 μg/kg/min;^d^ >0.05 μg/kg/min, ^e^Propofol or midazolam;^f^Evaluation out of sedation.

Pulmonary infection was the source of infection in the majority of septic patients (60%). Gram-negative pathogens were responsible for sepsis in 50% of septic patients. Twelve (66%) septic patients presented with one or more organ dysfunctions and (61%) had deterioration in their level of consciousness (GCS < 14).

**Figure 1:**
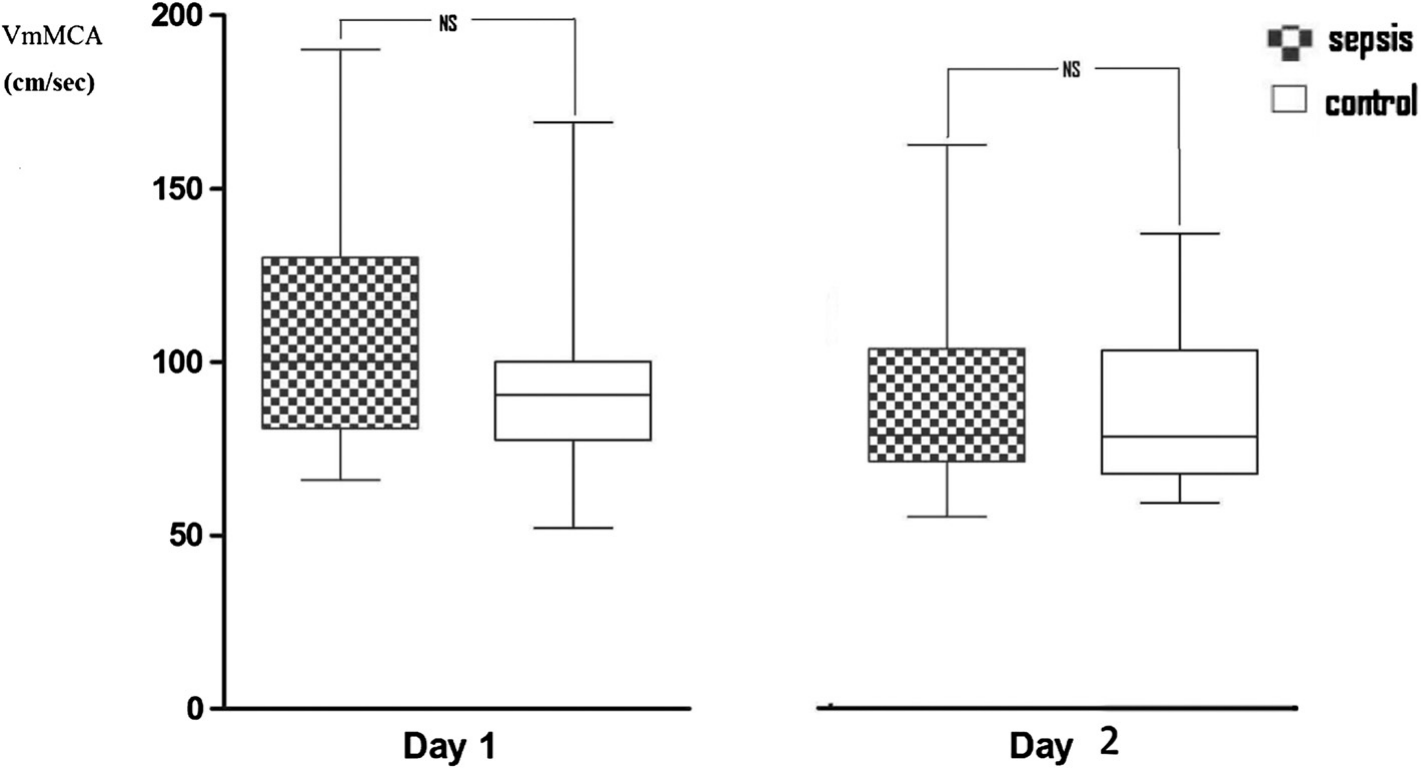
Mean velocity in middle cerebral artery (VmMCA) on the 2 days.

**Figure 2:**
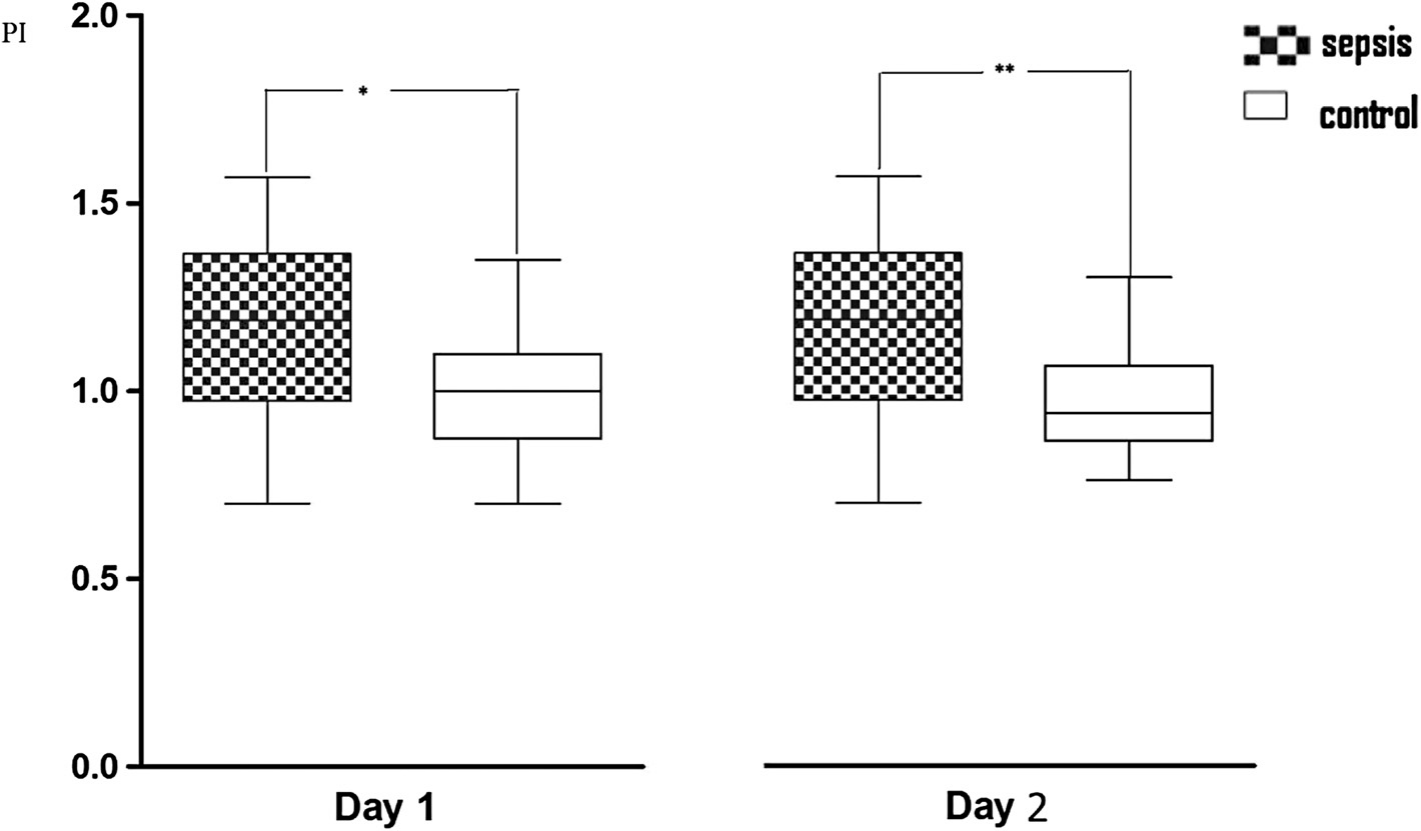
Pulsatility index (PI) on the 2 days.*p = 0.04; **p = 0.01.

**Figure 3:**
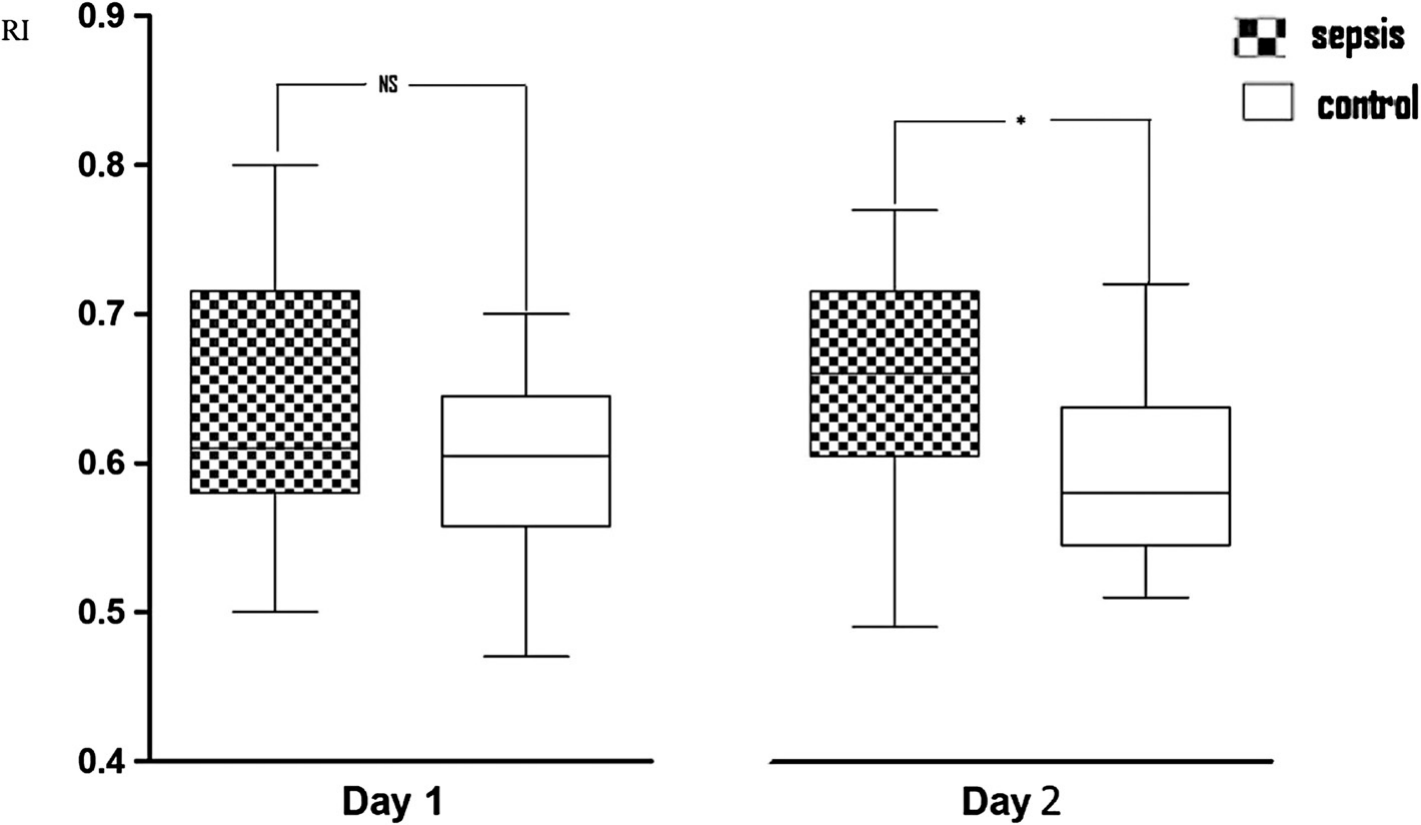
Resistance Index (RI) on the 2 days.

The measurements of VmMCA and calculations of PI, RI, and CBFi are presented in Tables 2, 3 and 4. Patients with sepsis did not have statistically significant higher values of VmMCA compared with nonseptic patients (110 ± 35 vs. 99 ± 28, *p* = 0.17). No differences of CBFi were found (497 ± 116 vs. 548 ± 110; *p* = 0.06). PI and RI were higher in patients with sepsis compared with nonseptic patients (1.15 ± 0.25 vs. 0.98 ± 0.16, *p* < 0.01, and 0.64 ± 0.08 vs. 0.59 ± 0.06, *p* < 0.01, respectively). The evolution of VmMCA, PI, and RI over the 2 days of observation is presented in Figures 1, 2, and 3. No correlation was found between VmMCA or PI or RI and MAP or pCO_2_ in either of the two groups (Table 5). No correlation of VmMCA and CBFi was found.

**Table 2:** Pooled data (days 1 and 2) of Transcranial Doppler measurements for the two groups of patients

	**Sepsis group**	**Control Group**	**p values**
No. measurements	36	36	
VMCA systolic (cm/sec)	192 ± 59	166 ± 51	0.05
VMCA diastolic (cm/sec)	68 ± 26	67 ± 18	0.83
VMCA mean (cm/sec)	110 ± 34	99 ± 28	0.17
Pulsatility Index (PI)	1.15 ± 0.25	0.98 ± 0.16	<0.01
Resistance index (RI)	0.64 ± 0.08	0.59 ± 0.06	<0.01
CBFi	497 ± 116	548 ± 110	0.06

*VMCA*, velocity in middle cerebral artery; *CBFi*, cerebral blood flow index.

**Table 3:** Day 1 data of Transcranial Doppler measurements for the two groups of patients

	**Sepsis group**	**Control Group**	**p values**
VMCA systolic (cm/sec)	189 ± 13	153 ± 12	0.05
VMCA diastolic (cm/sec)	67 ± 6	61 ± 3	0.32
VMCA mean (cm/sec)	106 ± 7	91 ± 5	0.11
Pulsatility Index (PI)	1.15 ± 0.06	0.99 ± 0.04	0.04
Resistance index (RI)	0.64 ± 0.02	0.60 ± 0.01	0.17
CBFi	469 ± 115	521 ± 113	0.513

*VMCA*, velocity in middle cerebral artery; *CBFi*, cerebral blood flow index.

**Table 4:** Day 2 data of Transcranial Doppler measurements for the two groups of patients

	**Sepsis group**	**Control Group**	**p values**
VMCA systolic (cm/sec)	195 ± 15	179 ± 12	0.44
VMCA diastolic (cm/sec)	68 ± 6	74 ± 5	0.53
VMCA mean (cm/sec)	112 ± 8	108 ± 7	0.71
Pulsatility Index (PI)	1.16 ± 0.06	0.96 ± 0.03	0.01
Resistance index (RI)	0.65 ± 0.01	0.58 ± 0.01	0.01
CBFi	498 ± 120	576 ± 104	0.05

*VMCA*, velocity in middle cerebral artery; *CBFi*, cerebral blood flow index.

**Table 5:** **Correlations of mean velocity in middle cerebral artery (VmMCA), pulsatility index (PI), resistance index (RI) and mean arterial pressure (MAP), pCO**_**2**_

	**Sepsis Group**				**Control Group**			
	**MAP**		**pCO**_**2**_		**MAP**		**pCO**_**2**_	
	**r**^**2**^	**p**	**r**^**2**^	**p**	**r**^**2**^	**p**	**r**^**2**^	**P**
VmMCA	0.01	0.95	0.01	0.48	0.01	0.93	0.00	0.96
PI	0.04	0.21	0.00	0.99	0.01	0.45	0.03	0.752
RI	0.04	0.23	0.00	0.98	0.01	0.43	0.01	0.53

## Discussion

The most important finding of this study is that TCD can be used in the evaluation of cerebral perfusion in septic patients. It is a noninvasive and feasible exam, because only two septic patients were not suitable for assessment as a result of an unsatisfactory echographic window. PI and RI were found to be increased in the sepsis group, suggesting early changes in cerebral perfusion during sepsis, as all of our patients were in sepsis for less than 48 hours.

Previous TCD studies performed in septic patients have focused on cerebral vasoreactivity in response to acetazolamide administration [12,13] or carbon dioxide [14,15] and blood pressure [16] modifications, with conflicting results. In contrast, static measurements of VmMCA were performed in our study with the calculation of PI as well as RI, and gross estimation of cerebral blood flow was made.

RI and PI are two parameters that are commonly used to describe the Doppler wave [22]. In vitro and in vivo studies show that both indexes are related to changes in peripheral vascular resistances [23-25]. Increased PI is associated with angiographically demonstrated diffuse intracranial vessel disease [26], and both RI and PI have been found to be increased in patients who suffer from diabetes with microangiopathy or advanced cirrhosis with encephalopathy [27,28]. The increased values of PI and RI that we observed in septic patients (compared with control critically ill patients) should be interpreted as a manifestation of enhanced vascular resistance in the cerebral circulation during early sepsis. This observation is consistent with previous experimental data documenting impaired cerebral microcirculation at the onset of septic shock [29] as well as in septic patients with delirium, where PI was found to be increased compared with healthy controls [13]. In this study, in contrast, we compared septic patients to a control group of critically ill patients to exclude critical illness-related factors (possibly including sedation, nonseptic inflammatory reaction, operation stress or catecholamines administration) that could affect cerebrovascular resistances. Patients from the control group had a similar PI to healthy people of the same age (0.98 ±16 vs. 0.95 ± 17 [30] or 0.97[0.93-1.02] [31]). Interestingly, on the first day, when microcirculation changes are expected to be less severe [29,32], only PI and not RI was found to be statistically higher in septic patients compared with the controls. This may be explained by the fact that PI seems to have greater sensitivity to reflect changes in cerebrovascular resistances than RI [33,34]. On the other hand, the results of our study support the hypothesis that changes in cerebral microcirculation start early in sepsis, whereas they are more well identified several hours after sepsis onset (>24 h) (Tables 3 and 4).

In our study, septic patients had higher arterial pCO_2_ than control patients. Acute hypercapnia normally causes intense cerebral vasodilation if vascular autoregulation is preserved. It therefore seems unlikely that higher pCO_2_ could account for the changes in vascular resistances that we observed in our septic patients, because any modifications would have been expected to be in the opposite direction. Cerebral autoregulation, however, has been shown to be influenced by carbon dioxide levels in patients with septic shock, with more profound alterations in autoregulation detected in hypercapnic (pCO_2_ > 40 mmHg) than hypocapnic (pCO_2_ < 40 mmHg) patients [14]. The relative vasodilation that was expected in our septic group (due to higher PaCO_2_) may thus have been partly blunted in some patients due to severe sepsis; however, this effect, if present, was only marginal, because we did not find any correlation between the levels of pCO_2_ and PI or RI (Table 5).

**Figure 4:**
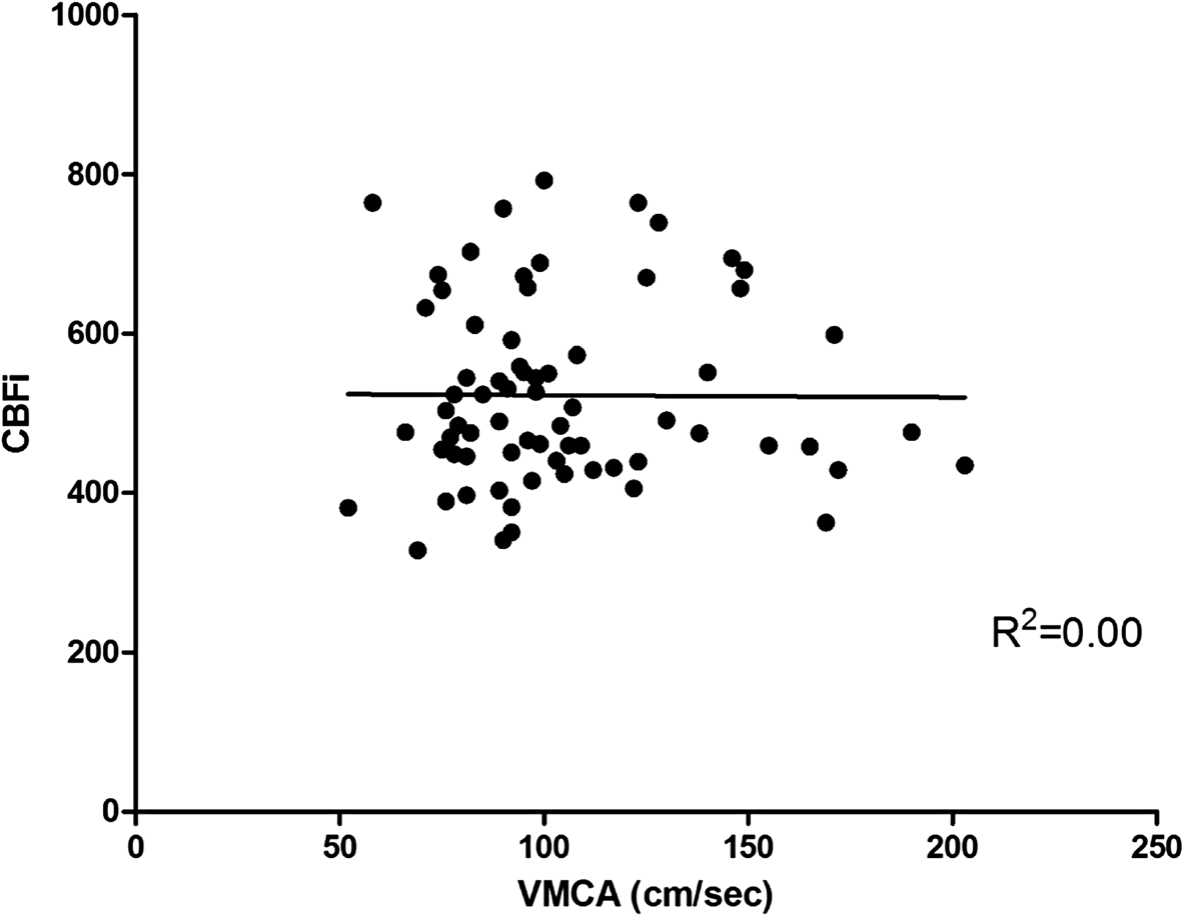
Correlation between cerebral blood flow index (CBFi) and mean velocity in the middle cerebral artery (VmMCA, cm/sec).

Enhanced cerebrovascular resistance (evidenced by elevated PI and RI) may expose septic patients to a decrease in cerebral blood flow (CBF) if not compensated by an increase in cerebral perfusion pressure (CPP). Unfortunately, to date, there is no method to measure cerebral perfusion in clinical practice and we cannot be sure whether there is any decrease of CBF in sepsis [9]. Assuming normal intracranial pressure in our patients and based on previous experimental data, we introduced a new index for a gross evaluation of CBF. According to this index, we did not find a statistically significant difference of CBF between the septic patients and controls. This can possibly be explained by the fact that the septic patients were evaluated at stable hemodynamic conditions with MAP that was not statistically significant lower than controls. Changes of cerebral microcirculation and not the actual CBF may potentially play a pivotal role in the pathophysiology of SAE. Apparently, this formula is not validated and our results should be considered cautiously. Even though PI is expected to be related to cerebral resistances linearly, that remains a hypothesis that should be further evaluated [21,22]. However, we believe that this index should be further assessed as it is not related to VMCA (Figure 4), which possibly indicates that CBFi gives different information to VMCA and does not seem to be dependent on the insonation angle applied by the performer.

Our study entails several limitations. First, the study was not blinded and the number of patients was relatively small; our results should thus be confirmed in a larger study with an ultrasonographer blinded for the presence of sepsis. A second theoretical limitation of our study relates to the possible operator-dependency and subjectivity of the ultrasound measurements. It is well established, however, that PI and RI measurements do not depend on the insonation angle applied by the performer [35]. Also, we did not repeat the TCD studies beyond the two consecutive study days; therefore, we do not know whether the observed TCD abnormalities were transient or persisted throughout the sepsis period. Finally, as far as SAE is concerned, the relatively small number of our study patients precludes any attempt to correlate neurological symptoms with TCD measurements or derived parameters. However, the higher number of septic patients with abnormal GCS (GCS < 14) may suggest a relation between our findings and clinical signs of SAE.

## Conclusions

Our results suggest that cerebral vascular constriction is detectable by TCD in the early stage of sepsis. TCD can be a useful tool to evaluate cerebral vascular tone and possibly cerebral perfusion in critically ill septic patients. Further studies are warranted to confirm the increases in PI and RI in sepsis patients and to assess the clinical significance of these results.

## Competing interests

The authors declared that they have no competing interests.

## Authors’ contributions

CP designed the study and collected the data. MO & AD contributed to conception and design. CP drafted the manuscript. CP, AA, DV, PB, PE, IM, MO, AD contributed to interpretation of the data and revision of the manuscript. All authors read and approved the final manuscript.
